# Systematic reviews of adverse effects: framework for a structured approach

**DOI:** 10.1186/1471-2288-7-32

**Published:** 2007-07-05

**Authors:** Yoon K Loke, Deirdre Price, Andrew Herxheimer, the Cochrane Adverse Effects Methods Group

**Affiliations:** 1 School of Medicine, Health Policy and Practice, University of East Anglia, Norwich, NR4 7TJ, UK; 2 Department of Clinical Pharmacology, University of Oxford, Radcliffe Infirmary, Woodstock Road, Oxford, OX2 6HE, UK; 3 The UK Cochrane Centre, NHS R&D Programme, Summertown Pavilion, Middle Way, Oxford, OX2 7LG, UK

## Abstract

**Background:**

As every healthcare intervention carries some risk of harm, clinical decision making needs to be supported by a systematic assessment of the balance of benefit to harm. A systematic review that considers only the favourable outcomes of an intervention, without also assessing the adverse effects, can mislead by introducing a bias favouring the intervention.

Much of the current guidance on systematic reviews is directed towards the evaluation of effectiveness; but this differs in important ways from the methods used in assessing the safety and tolerability of an intervention. A detailed discussion of why, how and when to include adverse effects in a systematic review, is required.

**Methods:**

This discussion paper, which presupposes a basic knowledge of systematic review methodology, was developed by consensus among experienced reviewers, members of the Adverse Effects Subgroup of The Cochrane Collaboration, and supplemented by a consultation of content experts in reviews methodology, as well as those working in drug safety.

**Results:**

A logical framework for making decisions in reviews that incorporate adverse effects is provided. We explore situations where a comprehensive investigation of adverse effects is warranted and suggest strategies to identify practicable and clinically useful outcomes. The advantages and disadvantages of including observational and experimental study designs are reviewed. The consequences of including separate studies for intended and unintended effects are explained. Detailed advice is given on designing electronic searches for studies with adverse effects data. Reviewers of adverse effects are given general guidance on the assessment of study bias, data collection, analysis, presentation and the interpretation of harms in a systematic review.

**Conclusion:**

Readers need to be able to recognize how strategic choices made in the review process determine what harms are found, and how the findings may affect clinical decisions. Researchers undertaking a systematic review that incorporates adverse effect data should understand the rationale for the suggested methods and be able to implement them in their review.

## Background

Systematic reviews are key components in the evidence-based medicine movement but these reviews have up till now concentrated on determining the effectiveness of healthcare interventions. This emphasis on treatment benefit, together with omission of information on harmful effects, could misinform anyone trying to make balanced treatment decisions. It is now becoming clear that the harmful effects of interventions should be reviewed with similar rigour. We therefore believe that it is not acceptable in the vast majority of instances to systematically review only the beneficial effects of an intervention. In the rare case where harms are justifiably excluded, the reviewers should explain that decision.

Research on 256 systematic reviews of adverse effects has found substantial uncertainties in the methodology [1]. These problematic areas need to be empirically investigated, but meanwhile a framework is needed to guide reviewers through this uncertain and evolving area.

The structured approach we present here is based on the empirical advice given in the Cochrane Handbook for Systematic Reviews of Interventions. [2] This framework was developed across meetings and workshops of the Adverse Effects Subgroup of The Cochrane Collaboration from 2001–2004. The drafting of the manuscript was supplemented by a consultation of experts in the methods used in systematic reviews, as well as those working in drug safety (listed in the Acknowledgements).

## Methods

### Formulating the problem: rationale, context, structure, scope

#### Rationale

As with reviews of effectiveness, a clearly focused research question is essential in a review of adverse effects. Relevant questions will be those that are directly aimed at guiding the decisions of clinicians, consumers, researchers and policymakers. A protocol should be developed for the systematic review and details of the research question specified, including the types of participants, interventions/exposures, comparisons, outcomes, and study designs to be included. Selection of outcomes and study type requires careful consideration in reviews of adverse effects (See Scope). Systematic reviews which include adverse effects are likely to make more than one comparison. In that case the early stages of the protocol should specify what the main comparisons will be and whether the review will consider both beneficial and adverse effects. (See 'Using the research question to structure the review').

#### Context

A systematic review is time and resource intensive, more so when it includes adverse effects. The resources devoted to studying adverse/unintended outcomes should reflect the importance of the treatment in context. For instance, if a treatment confers little benefit and is seldom used, its adverse effects may not be worth detailed evaluation. Some interventions do require an exhaustive analysis of all harmful outcomes, but for others adverse effects require a less thorough investigation. [3] Table 1 describes some specific therapeutic situations which warrant a detailed evaluation of adverse effects.

**Table 1:** Scenarios where detailed evaluation of adverse effects may decisively influence the decision whether to use an intervention or not.

**The margin between benefit and harm is narrow**	**Examples:**
Treatment is of modest or uncertain benefit, with some possibility of harm	• Aspirin for prevention of cardiovascular events in a healthy patient. What is the increased risk of haemorrhage from aspirin?
Treatment potentially very beneficial, but there are major safety concerns	• Carotid artery surgery in elderly patients with ischaemic heart disease who have suffered a stroke. The operation can prevent a future stroke but some patients may die from the intervention.
Treatment potentially beneficial in long-term, or to community. No immediate direct benefit to individual but possibility of harm exists.	• Improving uptake of a childhood vaccine to promote population immunity, while trying to assuage individual parents' fears about early serious vaccine- induced neurological damage to their child.

**Several effective treatments have differing safety profiles – which should be preferred?**	**Examples:**

Treatments are similarly effective, but have different safety profiles	• A new insulin injection device is thought to cause less pain than the existing device
The balance of benefits and harms differ substantially, e.g. the most effective intervention may have serious adverse effects, while the less effective one is potentially safer	• Radical mastectomy for breast cancer as opposed to limited, breast-conserving surgery which is less disfiguring but may carry a greater risk of local cancer recurrence.

**Adverse effects with an important role in deterring a patient from continuing an effective treatment**	**Examples:**

Treatment offers important benefit but adverse effects threaten patient's adherence	• The options for a patient with severe heart failure who has responded well to an ACE inhibitor, but has persistent cough: stop the medication altogether, try a lower dose, or change to an angiotensin receptor blocker?

#### Using the research question to structure the review

Usually, when the focus of the research question is purely on safety and or tolerability, the effectiveness of the treatment is already known. For example, with MMR (measles, mumps and rubella vaccination), a review was designed to look at adverse effects alone. [4] However, in some instances reviewers intend to evaluate adverse effects as part of a combined review that also covers beneficial outcomes. For instance, the mortality-reducing impact of a new drug for breast cancer may be the main focus of a systematic review, but there is also a need to weigh up, at the same time, serious recent concerns about the drug causing heart failure. Reviews that aim to evaluate benefit and harm together will usually require a more complex design that can efficiently handle different sets of studies for various outcomes. Using different search strategies and/or eligibility criteria for studies of benefit and harm will generate two or more diverse groups of eligible studies.

#### Scope

Selection of adverse outcomes for the review can be difficult. Unlike reviews of effectiveness, where all beneficial outcomes are likely to be well recognized beforehand, specific adverse effects associated with a therapeutic intervention may be known in advance of the review, others will not. It may not be possible to specify in advance which effects will be most relevant to the review.

The research question about safety and tolerability in a review may be broad or narrow in scope. For example, a review with a broad scope might ask "what adverse effects are associated with antidepressant therapy in humans?" Or, a more narrowly focused review might examine the risk of suicide and suicidal behaviour in adolescents taking a serotonin reuptake inhibitor. Table 2 describes the advantages and disadvantages of addressing broad and narrow questions.

**Table 2:** Advantages and disadvantages of selecting a broad versus narrow research question for a systematic review of adverse effect

**Scope**	**Pros**	**Cons**
**Narrowly focused**, usually evaluating only a selected adverse outcome in detail. Example: Does antidepressant X increase the risk of suicides in teenagers?	Easiest approach, especially with regard to data extraction. Hypothesis-testing design allows reviews to focus on important adverse effects and reach conclusions about treatment decision [3]	Conclusions are limited to specific adverse effects, and do not provide a complete picture of the overall safety or tolerability profile. Method is appropriate only for adverse events known in advance
**Broad sweep**. Example: What common adverse effects might a patient experience when starting on a tricyclic antidepressant?	This method can evaluate new adverse effects that were previously unrecognized, and will provide a broad general view of potential problems. Can also be used as part of a scoping exercise to identify specific adverse events that merit a further, more detailed look using the narrow focused approach.	Danger of being swamped by vast quantities of heterogeneous data and of inappropriate pooling. Broad, non-specific evaluations can be resource-intensive and may yield a diverse amount of information from which it is difficult to draw any meaningful conclusions. Detection of previously unrecognized adverse effects may be better addressed through primary surveillance than in a systematic review [3]

In general, reviewers who have already identified important safety concerns (for instance, from the knowledge of the pharmacology, or anatomical site of the intervention) should carry out a narrow-focused evaluation covering particular aspects of the relevant adverse effects. On the other hand, reviewers who are not aware of any specific safety problems, could start with a general overview of the range of adverse effects associated with an intervention. A widely scoped review may be part of an initial evaluation which eventually throws up specific safety issues that merit further focused study.

Such scoping reviews need particular care during the protocol development. Whilst reviewers carrying out a narrow focused review may have to concentrate only on specific named adverse effects, those performing a broad review may be confronted with an unstructured mix of lists, tables and text covering many diverse adverse outcomes. This difficulty is compounded by the lack of consistency in reporting adverse effects and the absence of a common format for doing so. Conceptualizing an organizational framework for adverse outcomes at the protocol stage may help review authors to approach the data in a systematic, manageable and clinically useful way. A predefined classification of adverse effects, for example, as; diagnosed by clinician (e.g. gastrointestinal haemorrhage), diagnosed by laboratory results (e.g. hypokalaemia), or patient-reported symptoms (e.g. pain) will avoid the ad hoc prioritizing of selected adverse effects when reviewers are confronted with numerous adverse effects at the data collection stage.

### What types of studies?

No single recommendation is possible here, and any decisions have to be made case by case. The decision on what types of studies to include will depend primarily on the main focus of the research question, balancing the elements of type of adverse effects(s) of interest, rigour in searching, and time and resources available.

Good data on a well-recognized, easily detectable adverse effect may potentially be available from randomized clinical studies. In contrast, information on new, rare or long-term adverse effects are unlikely to be found in trial reports. Trial participants often differ from those given the intervention in everyday practice. Many RCTs set criteria which exclude patient groups who are potentially more likely to be harmed by adverse effects. The systematic evaluation of new or rare adverse effects may require the inclusion of other study designs: cohort, case-control, cross-sectional, and even case series.

On the other hand, authors planning to use such additional data sources should realise that estimates of the frequencies of adverse effects from published case reports and spontaneous reporting may differ greatly from the results obtained from a meta-analysis of double-blind, randomized controlled trials. [6] A study comparing adverse outcomes from randomized and non-randomized studies found that the latter often yield lower estimates of absolute risk of harm. [7]

### Locating and selecting studies

Authors need to develop a literature search strategy based on key elements in their research question: population, intervention (plus acceptable comparators), and outcomes. The review question determines the nature of the search strategy.

#### Conducting an adverse effects specific search

The optimal search strategy for specifically identifying reports of adverse effects has yet to be established. Two main approaches can be used, each with its own limitations; they are best combined to maximise sensitivity (the likelihood of not missing studies that might be relevant):

#### Searching electronic databases using index terms (also called controlled vocabulary or thesaurus terms)

Index terms such as MeSH (Medical Subject Headings) in MEDLINE and EMTREE in EMBASE are assigned to records in electronic databases to describe the studies. Subheadings can also be added to index terms to describe specific aspects – for example, side effects of drugs, or complications of surgery. Table 3 lists the index terms used to denote data on adverse effects in the major databases.

**Table 3:** Indexing terms in MEDLINE and EMBASE

**MEDLINE**	***EMBASE***
/adverse effects /poisoning /toxicity /chemically induced /contraindications /complications	/*side effect* /*adverse drug reaction* /*drug toxicity* /*complication*

*Example:* *Aspirin/adverse effects* *Here, Aspirin is the MeSH term and adverse effects is the subheading;*	*Example:* *Acetylsalicylic-acid/adverse-drug-reaction* *Acetylsalicylic-acid is the EMTREE term and adverse-drug-reaction is the subheading*.

Within a database, studies may be indexed in three different ways: (i) under the name of the intervention together with a subheading to denote that adverse effects occurred, for example, Aspirin/adverse effects, Mastectomy/complications; (ii) under the adverse event itself, together with the nature of the intervention, for example, Gastrointestinal Hemorrhage/and Aspirin/, Lymphedema/and surgery/; or (iii) occasionally only under the adverse event, for example, Hemorrhage/chemically-induced.

Thus, no single index or subheading search term can be relied on to identify all data on adverse effects, but a combination of index terms and subheadings will help to detect reports of major adverse effects which the indexers have considered significant. [8,9]

#### Searching electronic databases using free-text terms ('text words')

Terms used by authors in the title and abstract of their studies can be searched on databases of electronic records using free-text terms. Two problems seriously limit the value of free-text searching:

• authors use a wide range of terms to describe adverse effects, both in a general sense (toxicity, side-effect, adverse-effect) and more specifically (eg, lethargy, tiredness, malaise may be used synonymously). An author of a review may not know all these terms in advance but should try to include as many relevant synonyms as possible.

• the free-text search does not detect adverse effects not mentioned in the title or abstract of the study in the electronic record (even though they appear in the full report) [9]

A sensitive free-text search should incorporate this potentially wide variety of synonymous terms used to denote data on adverse effects in studies, while also taking into account different conventions in spelling and variations in the endings of terms. So it is necessary to include singular and plural terms. These terms used to describe adverse effects should then be combined with free-text terms used to describe the intervention of interest, for example (aspirin or acetylsalicylic acid) and (adverse or side or hemorrhage or haemorrhage or bleed or bleeding or blood loss).

Clearly no single approach will reliably yield all the studies that have data on adverse effects of an intervention. The search, therefore, needs to combine index terms and free-text terms and is likely to take several iterations. For instance, it may be necessary to repeat the electronic search incorporating additional index terms, subheadings and free-text terms derived from initially identified relevant studies. In deciding which combination of terms to use, authors will need to balance comprehensiveness (sensitivity) against precision. For example, an electronic search that retrieves 20,000 studies is likely to contain most of the relevant studies, but if only 300 (1.5%) are relevant it is very imprecise and resource intensive.

While a more specific and less onerous search can be performed using study design terms such as 'trial' or 'case-control', the disparate designs of safety evaluations (for instance, research using 'prescription event monitoring') and the different terms for describing non-randomized studies means that reviewers may miss potentially relevant data.

### Additional sources of information

Review authors planning an exhaustive search may wish to consider checking other sources of information on adverse effects, which include spontaneous reporting systems and data from regulatory agencies (Appendix B, Supplementary Materials, lists additional sources of information on harms.)

The value of including data from any of these additional sources is uncertain. For example, some cases in a spontaneous reporting system are inevitably spurious, and including lists of potentially false harms does not make the review more useful. While case reports of suspected adverse reactions are widely published in scientific journals, few of these reports have been subsequently investigated or confirmed to be valid. [10]

Moreover, a casual user cannot readily apply the detailed information on harms in large databases. Queries need to be made in the form of well-designed studies, projects separate from a systematic review.

### Assessing susceptibility to bias

There is often a major trade-off between the comprehensiveness and the quality of the adverse effects data included in a systematic review. Including evidence that is likely to be biased, even if no better evidence exists, may lead to biased conclusions. All included data should be critically discussed and rigorously appraised.

#### Assessment tools

Many tools exist for assessing methodological quality. [11] They should identify more rigorous studies with results closer to the 'truth' – presumably for both therapeutic and adverse effects. However, we lack empirical evidence for the relevance of quality tools to adverse effect analysis. Any available quality assessment tools should be used cautiously because they may apply only to the primary focus of the study – usually the beneficial effects of the intervention. For example, the treatment's benefits may have been studied in a placebo controlled, well-blinded, adequately concealed randomized trial, with standard laboratory measurements. In contrast, the adverse effects of the same treatment may be collected, when treatment allocation is known, via a self-assessment questionnaire completed only by a small proportion of participants. Although the primary portion of the study may be of high quality, the monitoring of the harmful effects of the treatments is not.

One helpful approach may be to categorize the data based on the study designs included in the analysis. For instance, reviewers could usefully point out that there was only small-scale observational evidence for a particular adverse outcome, while a different adverse effect was more comprehensively evaluated by specific monitoring in randomized studies.

#### General principles

Whatever instruments are selected for assessing bias in experimental and observational studies of adverse effects authors should consider two important aspects:

• How rigorous were the methods used to detect adverse effects?

• How good is the reporting?

Examples of potentially useful questions in each area are:

##### On conduct

Are definitions given of reported adverse effects?

How were adverse effects data collected: prospective/routine monitoring, spontaneous reporting, patient checklist/questionnaire/diary; systematic survey of patients?

##### On reporting

Were any patients excluded from the adverse effects analysis?

Did the report give numerical data by intervention group?

Which categories of adverse effects do the investigators report?

Did the investigators report on all important or serious adverse effects, and how were these defined?

Were the methods used for monitoring adverse effects reported?

Finally, non-randomized studies are prone to biases, which can be hard to identify and handle. So far only limited empirical guidance exists on how to avoid systematic bias. [12] The Newcastle-Ottawa scale for observational studies is one example of a tool which has been used in a few Cochrane reviews to assess potential biases. [13]

#### Detection methods

The methods used to monitor or detect adverse effects greatly influence adverse effect frequencies, and may lead to heterogeneity in systematic reviews. For example, active surveillance and use of checklists have been shown to yield substantially higher frequencies of adverse events than the use of passive or less-focused methods. [14,15] Studies in which adverse effects are carefully sought will report a higher frequency than studies in which they are sought less carefully. Different methods of monitoring adverse effects yield different results, which may make comparisons between studies, let alone a formal meta-analysis, meaningless. [16]

#### Incomplete reporting

Incomplete reporting of results is also a particular problem with adverse effects. [17,18] For example:

• Certain categories **only **may be reported [e.g. the study states that events were defined by: several body systems, methods of collection, time periods (3, 6, 12 months), dose (20 mg, 40 mg, 80 mg), but the authors report only laboratory results for neurological disorders after 6 months with the 40 mg dose].

• Adverse event categories may not be clearly defined (for example, 'system = cardiovascular' but without indicating seriousness, severity, duration, diagnostic method, or final outcome).

• Treatment groups may be combined (for example, 'x participants withdrew from the study because of adverse effects').

• Generic statements (for example, 'no unexpected adverse effects were seen'/'there was no difference between the groups in adverse effects reported'/'the drugs were well tolerated').

In many instances (particularly with the generic statements above), greater account should be taken of what was left unsaid rather than what was actually reported.

### Collecting data

#### Terms

We suggest that information falling under any of the terms "adverse effect", "adverse drug reaction", "side effect", "toxic effect", "adverse event" and "complication" is potentially suitable for data extraction when evaluating the harmful effects of a treatment. (See Additional file 1, Appendix A, Definitions).

#### Exclusions

Note that no mention of adverse effects does not necessarily mean that no adverse effects occurred. It is usually safest to assume that they were not ascertained or not recorded: authors must choose whether to exclude the study from the adverse effect analysis or, exceptionally, to include it on the assumption that the incidence was zero.

#### Outcome characteristics

The definition of a particular adverse effect may vary between studies, as can definitions of severity. Moreover, a particular adverse effect may be described and/or measured in different ways among the trials – take for example, tiredness, fatigue or lethargy, all of which might be terms used in adverse effects reports. Authors may also use different thresholds for 'abnormal' results (for example, hypokalaemia diagnosed at a serum potassium concentration of 3.0 mmol/l or 3.5 mmol/l).

Are the adverse effects terms comparable across studies? Authors will need to decide which categories are similar enough to be lumped together in the analysis.

A number of initiatives aim to harmonise adverse effects terms. Examples include the National Cancer Institute severity grading system [19] and the WHO system-organ class categories. [20]

Review authors have used the WHO system to collate adverse effect data into one of several system-organ classes such as 'gastrointestinal system disorders'. [21] However, some researchers have found that attempts to shoehorn an adverse event report into the standard 'preferred terms' used by regulators and industry can distort descriptions in the original reports of adverse events and blur distinctions between them. [22]

#### Withdrawal or drop-outs as outcome measure

These outcome measures are often seen in trial reports. Caution is urged when interpreting such data as surrogate markers for safety or tolerability because of the potential for bias:

• The attribution of reason(s) for discontinuation is complex and may be due to mild but irritating side effects, serious toxicity, lack of efficacy, non-medical reasons, or a combination of causes. [23]

• Patients in trial conditions, and investigators, may have different thresholds in deciding whether to continue or end participation in the trial, and this may not reflect the true experience of adverse events within the general population. For instance, participants who have only limited access to medical facilities may be favourably disposed towards the enhanced follow-up they receive in a trial, and may decide to continue with the intervention, despite having adverse effects. Investigators who receive incentives based on the numbers of participants completing a trial may similarly be reluctant to withdraw patients with adverse effects. In both instances, the converse may also be true. For example, participants who have good access to high-quality medical facilities may, in the face of mild adverse effects, tend to end their trial participation early so that they can try out other options.

Unblinding of treatment assignment can precede the decision to withdraw. This can lead to an overestimate of the intervention's effect on patient withdrawal. For example, participants in the placebo arm who develop symptoms may be told that it is unrelated to their trial intervention, and are thus advised not to drop out. Conversely, patients in the active intervention group who complained of symptoms suggesting an adverse effect would be more readily withdrawn.

##### Quality of Life indicators

These are usually general measures which do not look specifically at particular adverse effects of the intervention. Quality of life scales can be used to gauge overall well-being, but they cannot be substituted for a detailed evaluation of safety and tolerability.

### Analysing and presenting results

If different types of studies are being used to evaluate beneficial and harmful effects, then an author must consider how to present potentially disparate datasets where studies reporting intended effects differ from those that report adverse effects. Special techniques have been devised in attempts to synthesise data from a diverse range of sources. [24,25]

The analysis of zero events in either arm (for example, "the drug was safe", and "no serious adverse effects were seen") needs careful consideration. Two questions in particular need to be asked:

• How thorough were the methods used to detect adverse effects?

• How many patients were studied and for how long?

One cannot conclude from 'zero events detected' that an intervention does not cause a suspected adverse effect. However, we can use the rule of three (for sample sizes > 30), to estimate the upper limit of the 95% confidence interval for the probability of the adverse effect. [26] If no events were detected in *n *trial participants, the upper limit of the 95% confidence interval for the probability of the adverse event is 3/*n*. In effect, this gives us a good idea of what the worst-case scenario could be, if no adverse events have occurred so far in our sample. If, for example, no adverse effects occur in 300 participants, then any adverse effects associated with the intervention might be as frequent as 1 in 100, but are unlikely to be more frequent. Note that studies with no events in either arm can be included in a meta-analysis of risk differences, but they cannot be included in a meta-analysis of odds ratios or risk ratios.

It is important to remember that a systematic review is not synonymous with a meta-analysis. In many circumstances adverse effect information is best summarised in a qualitative or descriptive manner. For instance, data derived from divergent sources cannot be combined because of different study design, different populations or, different data collection methods. It may not be possible to compare benefits and harms directly. In practice this means that adverse effects from RCTs, case reports, case series, cohorts, and case controls cannot all be pooled together using standard meta-analysis principles. Further, the data from non-randomised studies are more prone to bias, and are often heterogeneous; they should not be combined to produce a summary statistic if there is important heterogeneity.

Analysis and presentation of results categorized by study design can potentially provide useful insights into a particular adverse effect. In a systematic review evaluating pancreatitis with statins, data from case-control studies were pooled in a meta-analysis to yield a numerical estimate of the relative risk, and to allow assessment of statistical significance. [27] Data from case series and case reports were then used to identify important characteristics such as patients' age and gender, dose and duration of drug therapy, and other susceptibility factors involved in the adverse reaction.

### Interpreting results

#### Applicability

Many RCTs are restricted to selected subgroups of the population, and it is generally inappropriate to extrapolate adverse effects data from such studies to the wider population, which includes more vulnerable people, e.g. those with co-morbidities, co-medications. In interpreting adverse effects data, authors must consider the inclusion and exclusion criteria used in recruiting participants. Application of any finding to clinical practice will therefore always involve a degree of judgement.

#### Trade-offs

The assessment of benefit versus harm must be considered in the context of the nature of the condition for which the intervention is used. For instance, patients with life-threatening cancer are prepared to tolerate serious adverse effects from a potentially curative but disfiguring major surgical procedure. In contrast, patients with a self-limiting illness such as a sore throat may find that the inconvenience of having antibiotic-induced diarrhoea far outweighs the uncertain benefit of the drug therapy. These examples illustrate the need to evaluate the likelihood and magnitude of benefit and harm together, within context of the particular disease.

Similarly, the quality of evidence may also play a part in the benefit-harm trade-off. Where a treatment has been shown in randomized trials to have poor efficacy, even limited anecdotal evidence of possible adverse effects might make us decide against the intervention. However, a cancer patient who opts for a treatment of well-established efficacy would probably not be dissuaded by a few case reports of adverse effects.

Nevertheless, there may be difficulties in assessing the balance between benefits and harms when the analysis of harm includes studies beyond those included in the analysis of benefit. The patient populations used in the benefit and harm analyses may differ in important ways. Review authors will need to consider how much, if at all, the participants in the additional studies can differ from those in the benefit studies, and remain comparable.

For example, in a study of the benefits and harms of aspirin used as an antiplatelet drug to reduce cardiovascular events, a review author might want to include in the adverse effect analysis a study in which aspirin was used as an antiplatelet drug to reduce scarring after mastectomy. Predefined inclusion criteria, other than indication for treatment (dose, duration of treatment, reporting of adverse effects), would need to be met. The decision to include the study or not should depend on whether there is evidence that these women differ systematically in their risk of gastrointestinal haemorrhage from people who take the drug to prevent cardiovascular problems. [28]

## Conclusion

Authors are encouraged to recognize that strategic decisions taken in the review process can greatly influence what harms are found, and so affect future clinical decisions. This guidance document should help authors explore the consequences of the choices they make in conducting the review, and also to warn of pitfalls. Better conducted and reported studies of harms are certainly needed; and improvements will help to make systematic reviews of harms more accurate. [23]

It is also clear that specific methodological research is urgently needed in those areas where substantial deficiencies or uncertainties pose the greatest difficulties for reviewers. [1]

## Competing interests

The author(s) declare that they have no competing interests.

## Authors' contributions

All three authors contributed substantially to the drafting, review and revision of the manuscript. A wide range of expert advice was received from the helpful researchers acknowledged below.

## Pre-publication history

The pre-publication history for this paper can be accessed here:



## Supplementary Material

Additional file 1 **Systematic Review Adverse Effects Appendices. Appendix A. Definitions. Table 4. Definitions of terms related to adverse outcomes**. A list of terms used to describe harmful effects of healthcare interventions. **Appendix B. Additional sources of information**. A list of additional resources for review authors who are planning an exhaustive search. Click here for file
