# Supplementary material for: Systematic reviews of adverse effects: framework for a structured approach
Source: BMC Med Res Methodol. 2007 Jul 5;7:32. doi: 10.1186/1471-2288-7-32 (PMC1933432; doi:10.1186/1471-2288-7-32)
Supplement: Additional file 1 — Systematic Review Adverse Effects Appendices. Appendix A. Definitions. Table 4. Definitions of terms related to adverse outcomes. A list of terms used to describe harmful effects of healthcare interventions. Appendix B. Additional sources of information. A list of additional resources for review authors who are planning an exhaustive search. [file 1471-2288-7-32-S1.doc]

**Appendix A. Definitions**

Many terms are used to describe harmful effects of healthcare interventions; several of them are defined in Table 1. [1] [2] Published papers often use the terms ‘adverse effect’, ‘adverse drug reaction’, ‘side effect’, ‘toxic effect’, ‘adverse event’ and ‘complications’ loosely and interchangeably.

**Table 4 - Definitions of terms related to adverse outcomes**

| Adverse event | An unfavourable outcome that occurs during or after the use of a drug or other intervention but is not necessarily caused by it. It can be defined as “any abnormal sign, symptom, or laboratory test, or any syndromic combination of such abnormalities, any untoward or unplanned occurrence (for example, an accident or unplanned pregnancy), or any unexpected worsening or improvement in a concurrent illness” [2] |
| --- | --- |
| Adverse effect | An adverse event for which the causal relation between the drug/intervention and the event is at least a reasonable possibility. This term applies to all interventions. |
| Adverse drug reaction (ADR) | A term used only with drugs. The terms ADR and adverse effect are otherwise used interchangeably [2] |
| Complication | A term widely used to describe adverse events following surgical and other invasive interventions. ‘Adverse event’ and ‘adverse effect’ can be considered synonyms |
| Seriousness and intensity (or severity) of the adverse effect | Often confused with seriousness, severity is better termed ‘intensity’. WHO terminology differentiates between the terms ‘serious’ and ‘severe’ in this way: ‘Serious’ refers to adverse effects that have significant *medical* consequences, eg lead to death, permanent disability or prolonged hospitalisation. A review should state whether ‘serious’ is defined in this way, or whether it also includes effects that the patient considers serious. In contrast, 'severe' refers to the intensity of a particular adverse effect. For example, a non-serious adverse effect, such as headache, may be severe, moderate or mild. |
| Side effect | This is one type of adverse effect. It is any unintended effect of a pharmaceutical product that occurs at doses normally used for therapeutic purposes in man and is related to the pharmacological properties of the drug. While some side effects may be harmful (and can thus be considered adverse effects), others may be beneficial. |
| Safety | This word usually refers to (the relative lack of) serious adverse reactions, such as those that threaten life, require or prolong hospitalization, result in permanent disability, or cause birth defects. But, serious, indirect adverse effects, such as traffic accidents, violence, and damaging consequences of mood change, can also be categorized by this term. They may or may not be detected in trials (depending on participant numbers, method of monitoring, and length of follow up), and data on such adverse effects may be available only from non-randomised studies. |
| Tolerability | A term that usually refers to medically less important (ie without serious or permanent sequelae) but unpleasant adverse effects of drugs. These include symptoms such as dry mouth, tiredness, etc, that can affect a person’s quality of life and willingness to continue the treatment. As these adverse effects usually develop early and are relatively frequent, RCTs may yield reliable data on their incidence. |

**References:**

1. Aronson JK, Ferner RE: **Clarification of terminology in drug safety**. *Drug Saf* 2005, **28**(10):851-870.

2. Edwards IR, Aronson JK: **Adverse drug reactions: definitions, diagnosis, and management**. *Lancet* 2000, **356**(9237):1255-1259.

### Appendix B. Additional sources of information

Review authors who are planning an exhaustive search may wish to consider checking the following sources:

- Standard reference books on adverse effects, Meyler’s Side Effects of Drugs and the Side Effects of Drugs Annuals, and screening the papers they summarise.
- Bulletins from regulatory agencies, for example:
  - in the UK, Current Problems in Pharmacovigilance (http://medicines.mhra.gov.uk/ourwork/monitorsafequalmed/currentproblems/cpprevious.htm)
  - in Australia, the Australian Adverse Drug Reactions Bulletin (http://www.tga.gov.au/adr/aadrb.htm)
  - and the European Public Assessment Reports from the European Medicines Evaluation Agency (http://www.emea.eu.int/#).

Authors can also apply to the WHO Uppsala Monitoring Centre (UMC; http://www.who-umc.org) for special searches of their spontaneous reporting database; this was for example done for melatonin. [25]

Information on the safety of medical devices and surgical interventions is also held by various organisations. Examples are:

- UK National Joint Registry, which records details of hip and knee replacement operations in England and Wales
- The Medical Devices section of the UK Medicines and Healthcare Products Regulatory Agency (<http://devices.mhra.gov.uk/>)
- the US Food and Drugs Administration runs MEDWATCH, (<http://www.fda.gov/medwatch/index.html>) which records adverse events related to drugs, biologics, medical devices and herbal supplements
